# Caudatin Inhibits the Proliferation, Invasion, and Glycolysis of Osteosarcoma Cells via the Wnt/*β*- Catenin Pathway

**DOI:** 10.1155/2022/4026688

**Published:** 2022-12-23

**Authors:** Yan Zhang, Zhizhong Sheng, Chi Su, Yuli Xia, Xiangfan Chen, Xiaobo Huang, Honglin Li, Changsheng Ma, Ligang Wang

**Affiliations:** Pediatric Orthopedics, Shenzhen Pingle Orthopedic Hospital, No. 30, Dongyuan Road, Futian District, Shenzhen City 518000, Guangdong Province, China

## Abstract

**Background:**

Caudatin is a steroidal glycoside with reported anticancer activity in a variety of studies. Nevertheless, the role and mechanisms of caudatin in osteosarcoma (OS) remain unclear. In this study, we explored the potential anticancer effects of caudatin in OS cells and investigated the underlying mechanisms.

**Methods:**

Both the CCK8 proliferation assay and flow cytometry were employed to evaluate cell proliferation and apoptosis. A transwell assay was applied to determine cell invasion ability. Besides, glycolytic capacity was examined by measuring glucose consumption, lactic acid production, as well as ATP production. A western blot was utilized to assess the protein levels of *β*-catenin, CyclinD 1, C-myc, HK2 (Hexokinase 2), LDHA (lactate dehydrogenase), as well as epithelial-mesenchymal transition (EMT)-related markers. The inhibitory effect of caudatin on tumor growth was investigated using a xenograft tumorigenesis model.

**Results:**

Caudatin restrained cellular glycolysis, suppressed cell proliferation and invasion by reducing HK2 and LDHA expression and regulating the Wnt/*β*-Catenin signaling pathway. Caudatin treatment caused the upregulation of E-cadherin and suppressed N-cadherin expression. Further, caudatin treatment impaired cell viability, invasion ability, and intracellular glycolysis level but induced apoptosis. The administration of BML 284 reversed the inhibitory effects of caudatin. Moreover, caudatin suppressed the tumorigenesis of OS cells in the xenograft model of nude mice.

**Conclusions:**

Our study revealed the anticancer effects of caudatin, including proliferation inhibition, cell invasion suppression, and glycolysis impairment. These effects seem to be executed through targeting the Wnt/*β*-Catenin signaling pathway. These data indicate that caudatin may be formulated as a potential therapeutic for osteosarcoma.

## 1. Introduction

Osteosarcoma (OS) is a malignant tumor that originates from the osteoblast precursor cells in the bones [1, 2]. OS usually occurs among adolescents in distal femur and proximal tibia, and its 5-year survival rate was as low as 20% [3]. At present, the optimal surgery supplemented by radiotherapy and chemotherapy remains the mainstay for OS treatment [4]. However, due to its high degree of malignancy and high incidence of metastasis, the prognosis of OS is very poor [5, 6]. Hence, it is imperative to develop novel therapeutics to restrain the proliferation and invasion of OS cells, which is of great significance for improving the treatment outcome and survival rate.

Caudatin is a C-21 steroidal glycoside extracted from the root of *Cynanchum* bungei Decne [7]. Previous studies have demonstrated that caudatin can induce cell apoptosis and impair cell invasion, and immune regulation is also implicated in its anticancer effect [8, 9]. LUO et al. reported that caudatin restrained cell growth and metastasis of human hepatoma cells by targeting cox-2 and mmp-2/9 [10]. TAN et al. demonstrated that caudatin could induce apoptosis in human uterine cancer cells via regulating the TNFAIP1/NF-*κ*B signaling axis [11]. Furthermore, Wang et al. revealed that caudatin regulated VEGF expression and promoted apoptosis through restraining the Wnt/*β*-Catenin pathway [12]. Nevertheless, the potential effects and mechanisms of caudatin in OS remain unclear.

In this study, we explored the inhibitory effects of caudatin in OS cells and investigated the underlying mechanisms. Our data provide insights into the anticancer activity of caudatin in OS cells, which may be developed into an alternative therapeutic strategy for osteosarcoma treatment.

## 2. Materials and Methods

### 2.1. Cell Culture

OS cell lines (U2OS and MG63) and normal cells were acquired from the cell resource center (Solarbio, China). U2OS and MG63 cells were cultured in DMEM medium containing 10% FBS (Solarbio, China). Cells were incubated in a humidified incubator at 37°C and 5% CO_2_.

### 2.2. The CCK-8 Proliferation Assay

A CCK-8 proliferation assay was utilized to determine cell viability. U2OS and MG63 cells (1 × 104 cells/well) were seeded in 96-well plates and treated with caudatin (15–150 *μ*M) for 24 h, 48 h, and 72 h. At the indicated time point, 10 *μ*L of CCK-8 solution (Solarbio, China) was added to each well of the 96-well plates. The cells were incubated at 37°C for 2 h, and the absorbance was recorded at 450 nm using a microplate reader (Solarbio, China).

### 2.3. Flow Cytometry

U2OS and MG63 cells (1 × 104 cells/well) were seeded in 96-well plates and treated with caudatin (15–150 *μ*M) for 48 h. Then, cells were incubated with Annexin V/PI staining solution (BD Biosciences, CA, USA) for 10 min in the dark. After the washing with PBS, cell apoptosis was examined by BD FACS CantoTM II Flow Cytometer (BD Biosciences), and the data were analyzed by Flowjo V10 software.

### 2.4. Glycolysis Level

U2OS and MG63 cells (1 × 104 cells/well) were seeded in 96-well plates and treated with caudatin (15–150 *μ*M) for 48 h. Then, cells were washed by PBS, lysed in RIPA buffer, and centrifuged at 10,000 × *g* for 10 min. The collected supernatant was saved at −80°C until measured. Glucose consumption, lactic acid production, and ATP level were measured using corresponding commercial kits (Solarbio, China) according to the manufacturer's instructions.

### 2.5. Transwell Invasion Assay

The Transwell assay was utilized to evaluate the ability of cell invasion. U2OS and MG63 ells in serum-free medium were inoculated in an upper chamber coated with Matrigel (Corning, CA, USA). The complete medium with 10% FBS was added into the lower chamber. After 24 h, cells were fixed with 4% paraformaldehyde (PFA) for 15 min and stained with 0.5% crystal violet (Solarbio, China) for 30 min. The stained cells were photographed and counted using a phase-contrast light microscope (Leica, Germany).

### 2.6. Western Blot

Cells were lysed with RIPA buffer (Beyotime, China) for 15 min on ice and centrifuged at 10,000 x *g* for 15 min. The supernatant was collected, and the concentration of the protein sample was measured with the BCA kit (Beyotime, China). Protein samples were separated by electrophoresis and transferred from gel to PVDF membrane (Beyotime, China). 5% BSA in PBS was used to block the PVDF membrane, which was followed by the incubation with primary antibodies: anti-*β*-catenin (1 : 1000, ab32572, abcam), anti-CyclinD 1 (1 : 1500, ab141491, abcam), anti-C-myc (1 : 1000, ab32072, abcam), anti-HK2 (1 : 1500, ab273721, abcam), anti-LDHA (1 : 1000, ab290306, abcam), anti-E-cadherin (1 : 1000, ab233611, abcam), anti-N-cadherin (1 : 1000, ab254512, abcam), as well as anti-GAPDH (1 : 1500, ab8245, abcam) antibodies. The membrane was washed 3 times with TBST for 5 minutes each. After washing, the membrane was further incubated with HRP-linked secondary antibody (1 : 3000; #7047, Cell Signaling Technologies, MA, USA) at room temperature for 1 h. The protein bands were developed using the ECL protein detection kit (Solarbio, China) and analyzed with ImageJ software (NIH, USA).

### 2.7. Treatment of the Wnt/*β*-Catenin BML 284 (Wnt Agonist)

U2OS and MG63 cells were divided into control, caudatin (100 *μ*M), and caudatin (100 *μ*M) + BML 284 (20 mmol/L) groups. Cells were then subjected to the CCK-8 proliferation assay, flow cytometry analysis, glycolysis level determination, transwell invasion assay, and western blot analysis.

### 2.8. Immunohistochemistry (IHC) and Hematoxylin and Eosin (H&E) Staining

Tumor tissues were fixed with 10% formalin overnight and then embedded with paraffin. The tissue was cut into 4 *μ*m sections, and the sections were dewaxed for immunohistochemistry. H&E staining was performed using a commercial staining kit (ab245880, Abcam) according to the manufacturer's instructions. For IHC staining, the section was blocked with 5% BSA for 1 h and then incubated with anti-Ki-67 antibodies (1 : 1000, ab270650, abcam) overnight. After washing with TBST buffer, the section was soaked with 2 drops of SignalStain® Boost Detection Reagent (HRP, Rabbit #8114, Cell Signaling Technologies) for 30 min at room temperature. Signal development was performed for 5 min using SignalStain® Substrate (#8059, Cell Signaling Technologies). The images were captured using a phase-contrast light microscope (Leica, Germany).

### 2.9. Xenograft Tumorigenesis in Nude Mice

To evaluate the in vivo anticancer action of caudatin, mice (4–6 weeks old immunodeficient Balb/C nude mice, Shanghai, China) were injected with MG63 cells (5 × 106) to establish xenograft tumorigenesis model, according to the published report [13]. 2 weeks after cell injection, mice (*n* = 12) were randomly divided into sham (intraperitoneal injection with PBS) and caudatin (intraperitoneal injection with 50 mg/kg every three days). The volume of the tumor was recorded every 6 days using a caliper, and the tumor volume was calculated using the formula V(tumor) = 0.5 × length × width2. On day 30, the mice were sacrificed, and the tumors were removed and weighed. In addition, the tissues were fixed with 4% paraformaldehyde and stored at −80°C for immunohistochemical assay. All the above animal operations followed the approval of the ethics committee.

### 2.10. Statistical Analysis

Experimental data were shown by mean ± SD. The differences between the control and caudatin treatment groups were analyzed by one-way analysis of variance (ANOVA) or unpaired Student's *t*-test. *P* < 0.05 was considered a significant difference.

## 3. Results

### 3.1. Caudatin Restrained Cell Proliferation and Induced Apoptosis

**Figure 1:**
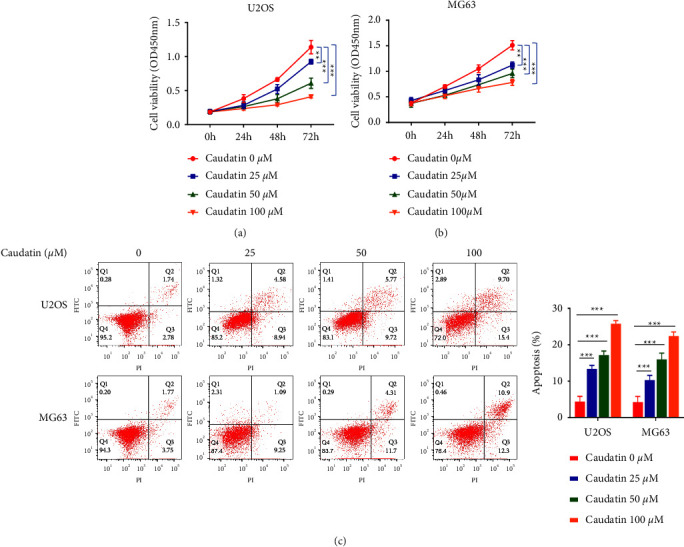
Caudatin restrained cell proliferation and promoted apoptosis. (a–b) U2OS and MG63 cells are treated with caudatin (15–150 *μ*M) for 24, 48, and 72 h, and cell proliferation is evaluated by the CCK-8 cell proliferation assay. (c) Apoptosis in caudatin-treated OS cells via a flow cytometer. ^*∗*/*∗∗*^*P* < 0.05/0.01, compared with con, ^#/##^*P* < 0.05/0.01, compared with caudatin (25 *μ*M) *p* < 0.01.

Firstly, we analyzed the anticancer effects of caudatin (25–150 *μ*M) on cell growth in U2OS and MG63 cells via the CCK-8 proliferation assay at different time points (24, 48, and 72 h). The results in Figures 1(a) and 1(b) showed that caudatin (25, 50, and 100 *μ*M) restrained the proliferation of OS cells in a dose/time-dependent manner, and the growth inhibition effect was evident at 48 hours. Hence, we applied different concentrations of caudatin (25, 50, and 100 *μ*M) at 48 h in follow-up experiments. A flow cytometry-based Annexin V and PI staining assay were utilized to evaluate the apoptosis of cells. The results showed that caudatin treatment induced apoptosis in a dose-dependent manner in both the U2OS and MG63 cells (Figure 1(c)).

### 3.2. Caudatin Inhibited the Invasion of OS Cells

**Figure 2:**
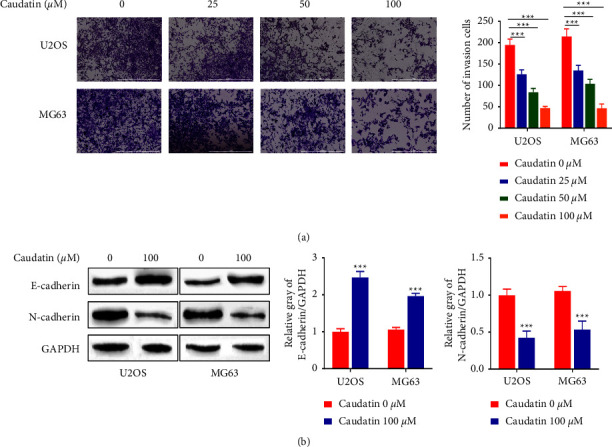
Caudatin inhibited the invasion of OS cells. (a) The invasion abilities of caudatin-treated U2OS and MG63 cells are measured via a transwell invasion assay. (b) Western blot analysis of E-cadherin and N-cadherin expression in caudatin-treated cells. ^*∗*/*∗∗*^*P* < 0.05/0.01, compared with con, ^#/##^*P* < 0.05/0.01, compared with caudatin (25 *μ*M).

We carried out a transwell invasion assay, in order to investigate the anti-invasion effects of U2OS and MG63 cells. Results in Figure 2(a) demonstrate that caudatin significantly hindered cell invasion in a dose-dependent manner (*p* < 0.05, *p* < 0.01). Since the invasion of cells was closely related to the epithelial-mesenchymal transition (EMT) process, we detected the changes in EMT-related proteins upon caudatin treatment. Results in Figure 2(b) show that caudatin (100 *μ*M) increased E-cadherin expression (epithelial marker) and reduced N-cadherin expression (mesenchymal marker) (*p* < 0.01).

### 3.3. Caudatin Restrained Glycolysis of OS Cells

**Figure 3:**
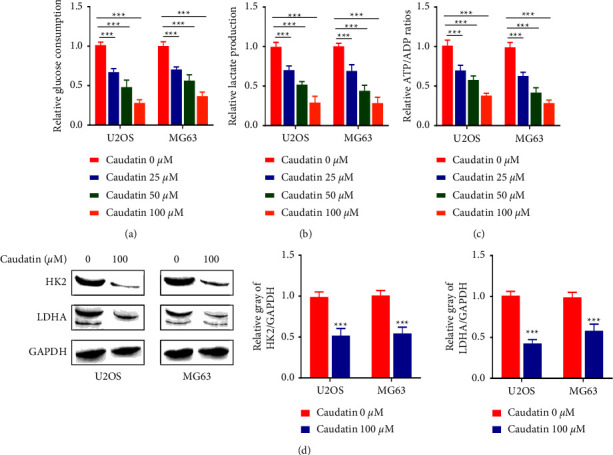
Caudatin restrained glycolysis of OS cells. (a–c) The glucose consumption, lactic acid production, and ATP levels of U2OS and MG63 cells are measured with or without caudatin treatment. (d) Western blot analysis of HK2 and LDHA expressions in caudatin-treated cells. ^*∗*/*∗∗*^*P* < 0.05/0.01, compared with con, ^#/##^*P* < 0.05/0.01, compared with caudatin (25 *μ*M).

Glycolysis is an important metabolic process in cancer cell proliferation, and the inhibition of glycolysis was able to restrain proliferation and cause cell death. Results in Figures 3(a) and 3(c) demonstrate that caudatin markedly reduced glucose consumption, lactic acid production, and ATP production in a dose-dependent manner (*p* < 0.05, *p* < 0.01). Furthermore, we examined the key proteins HK2 (Hexokinase (2)) and LDHA (lactate dehydrogenase) involved in glycolysis and lactate production. Western blot results showed that caudatin treatment significantly reduced the protein levels of HK2 and LDHA U2OS and MG63 cells (Figure 3(d), *p* < 0.01).

### 3.4. Caudatin Inhibited the Wnt/*β*-Catenin Pathway in OS Cells

**Figure 4:**
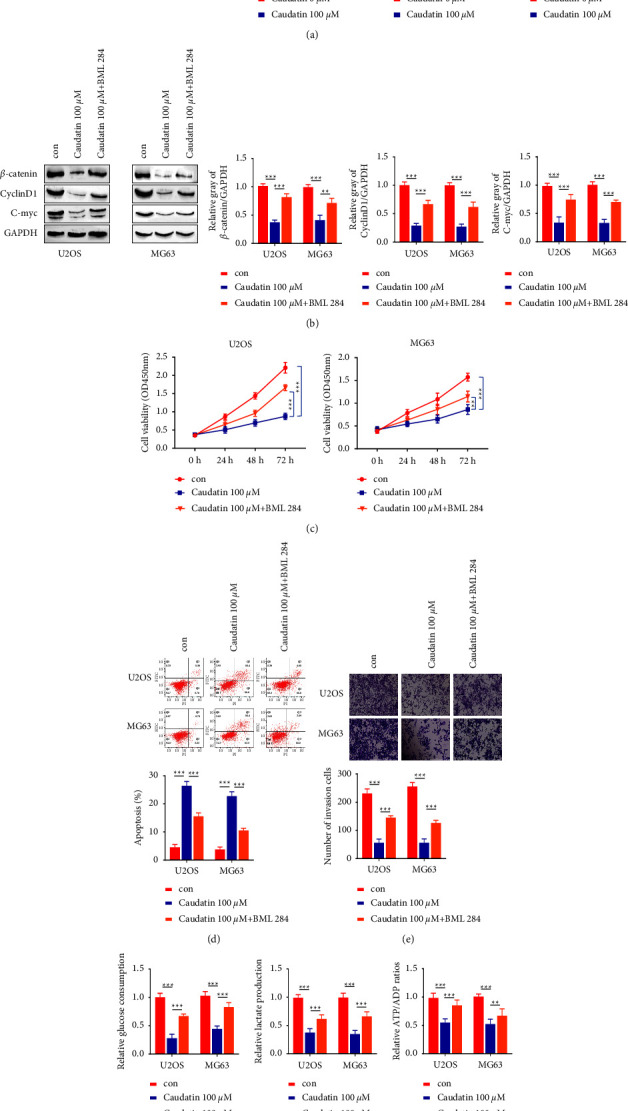
Caudatin inhibited the Wnt/*β*- Catenin pathway in OS cells. Cells are then divided into the control, caudatin, and caudatin + BML 284 (Wnt agonist) groups. (a–b) Western blot analysis of *β*-catenin, CyclinD 1, and C-myc protein levels in different groups. (c–f) Cell proliferation, invasion ability, cellular apoptosis, and different parameters of glycolysis in U2OS and MG63 cells are measured in different treatment groups. ^#/##^*P* < 0.05/0.01, compared with con, ^*a*/*aa*^*P* < 0.05/0.01, compared with caudatin (100 *μ*M).

The activation of the Wnt pathway is closely related to tumor metastasis, invasion, and drug resistance [14, 15]. We therefore investigated the expression of the *β*-Catenin as well as cell proliferation-related proteins (Cyclin *D* 1 and C-myc) upon caudatin treatment. Western blot results demonstrated that caudatin (100 *μ*M) markedly reduced *β*-catenin, Cyclin *D* 1, and C-myc expression in U2OS and MG63 cells (Figure 4(a), *p* < 0.01), suggesting that caudatin might exert its anticancer effects via the Wnt/*β*-Catenin pathway. To further verify this finding, cells were then divided into the control, caudatin treatment, and caudatin + BML284 (Wnt agonist) groups. Western blot results demonstrated BML284 treatment attenuated the effects on the expression of *β*-catenin, Cyclin *D* 1, and C-myc (Figure 4(b)). In addition, we investigated whether BML284 administration affected the anticancer effect of caudatin. Functional assays showed that BML284 treatment largely impaired the effects of caudatin on cell proliferation inhibition, apoptosis induction, and invasion suppression (Figures 4(c) and 4(d)). Furthermore, the treatment of BML 284 also partially reversed the inhibitory effects of caudatin on glucose consumption, lactic acid production, and ATP production (Figure 4(f)). These results indicate that the anticancer effects of caudatin on OS cells are mediated through the Wnt/*β*- Catenin pathway.

### 3.5. Caudatin Restrained the Tumorigenesis of OS Cells *in Vivo*

**Figure 5:**
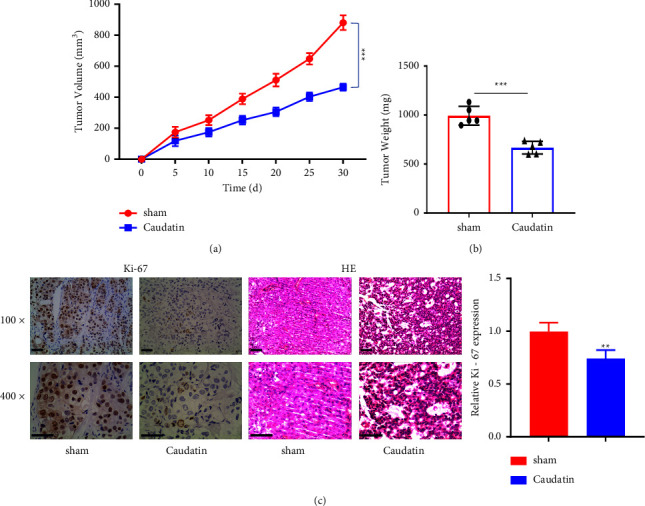
Caudatin restrained the proliferation of OS cells *in vivo*. MG63 cells are injected to nude mice, and 2 weeks after cell injection mice were randomly divided into the sham (injected with PBS) and caudatin (injected with 50 mg/kg every three days) groups. (a) Tumor volume and (b) tumor weight are measured in the sham and caudatin groups. (c) The expression level of Ki-67 is measured via immunohistochemistry. ^*∗*/*∗∗*^*P* < 0.05/0.01, compared with sham.

To further investigate the anticancer effects of caudatin on OS cells *in vivo*, a xenograft model of OS cells in nude mice was established. 2 weeks after cell injection, mice were randomly divided into the sham (injected with PBS) and caudatin (injected with 50 mg/kg every three days) groups. After 30 d administration, the tumor volume and weight were significantly reduced in the caudatin group (Figures 5(a) and 5(b)). Immunohistochemistry staining further showed that the expression of Ki-67 (proliferation marker) was largely reduced by caudatin administration (Figure 5(c)).

## 4. Discussion

Since osteosarcoma (OS) has a high incidence of recurrence and metastasis, therefore, intensive research has been focused on the mechanisms underlying its metastasis and invasion [16–19]. In this study, we first showed that caudatin could restrain the malignant phenotypes of OS cells by inhibiting cell proliferation, inducing apoptosis, and impairing cellular glycolysis. We further demonstrated that the anticancer effects of caudatin is related to the suppression of the Wnt/*β*-Catenin pathway.

Cancer cells are characterized by high malignancy, with augmented invasiveness and metastasis compared to normal cells [17]. Glucose and lactic acid are the raw materials and metabolites of aerobic glycolysis, which is the main metabolic support for cancer development [20, 21]. ATP is the major energy carrier supporting different cellular processes [23]. In addition, high levels of ATP and a low ratio of NAD + /NADH are the characteristics of the energy metabolism of tumor cells [24–26]. HK2 is the rate-limiting enzyme of the glycolysis pathway [27]. The reduced expression of HK2 can limit the capacity of glycolysis [28]. LDHA is the key enzyme involved in the production and transport of lactic acid, a glycolytic product [29, 30]. Song et al. discovered that si-PVT1 could downregulate HK2 expression, reduce glucose uptake, and lower lactate production in OS cells [31]. Han et al. demonstrated that the knockout of HK2 could inhibit the oncogenic role of TUG1 on OS glycolysis [32]. Besides, silencing LDHA could hinder the consumption of glucose and suppress the malignancy of OS cells [33]. Consistent with these studies, our data showed that caudatin represses glucose consumption by downregulating the glycolytic enzyme HK2, which results in the reduced production of lactic acid and ATP. Our data indicate that caudatin could not impair glycolysis and the energy supply to tumor cells, thus restraining the proliferation of OS cells. These data further imply that caudatin might exert its antitumor activity by regulating HK2 and LDHA expression in glycolysis, which may be a latent blocker of tumor energy metabolism.

The activation of the Wnt pathway is closely related to tumor metastasis, invasion, and drug resistance [14, 15]. Gomaa et al. reported that the deregulation of Wnt/*β*-Catenin, E-cadherin, and N-cadherin could lead to a poor prognosis in patients with colorectal cancer [33]. García-Cabo et al. also demonstrated that the expression level of *β*-Catenin is a risk factor for poor prognosis in laryngeal cell carcinoma [34]. Moreover, the inactivation of the Wnt/*β*-Catenin pathway promotes apoptotic cell death in human ovarian cancer cells [35]. In addition, chemical drugs inhibiting Wnt/*β*-Catenin pathway or small interfering RNA targeting *β*-Catenin expression could induce apoptosis [36]. Hence, we reasoned that caudatin inhibits the growth of OS cells by targeting the Wnt/*β*-Catenin pathway. This notion was supported by the observation that Wnt agonist (BML 284) could largely impair the anticancer effects of caudatin, including inhibiting proliferation and invasion ability and impairing intracellular glycolysis and promoting apoptosis. These results further corroborate that caudatin abrogates the aggressive phenotypes of OS cells by inhibiting the Wnt/*β*-Catenin pathway.

In conclusion, we demonstrated that caudatin could restrain the proliferation, glycolysis, and invasion ability of OS cells by targeting the Wnt/*β*- Catenin pathway. Importantly, caudatin administration impairs the tumorigenesis of OS cells in the xenograft mouse model. These data suggest that caudatin may be formulated as a therapeutic strategy for osteosarcoma.

## Data Availability

The raw data supporting the conclusion of this article will be made available by the corresponding author without undue reservation.
